# The Chicago Gynæcological Society: Meeting June 18

**Published:** 1886-09

**Authors:** 

**Affiliations:** 2330 Indiana Ave.


### TRANSACTIONS OF THE CHICAGO GYNÆCOLOGICAL SOCIETY.

*Regular Meeting, Friday, June 18, 1886.*

*I.*—JAGGARD. *A Gravid Uterus with Adnexa, Corresponding to the Sixth Month.*

*II.*—PARKES. *Discussion of the Paper on Uterine Fibroids Treated by the Fluid Extract of Ergot.*

*III.*—BYFORD. *A Study of the Causes and Treatment of Pelvic Hæmatoceles.*

The PRESIDENT, DANIEL T. NELSON, M. D., in the chair.

I.—PROFESSOR W. W. JAGGARD exhibited

### A GRAVID UTERUS WITH ADNEXA, CORRESPONDING TO THE SIXTH MONTH.

The material was placed at his disposal through the conrtesy of Dr. H. H. Frothingham, one of the resident obstetricians of Cook County Hospital.

The patient, 30 years old, multipara, came under observation May 17, 1886. While sitting on a chair in the ward she began to show signs of asphyxia. She was immediately put to bed, but died within five minutes of the beginning of the attack.

*Autopsy* made forty-eight hours after death.

*External Appearances.*—Some venous hypostasis over dependent portions.

*Lungs.*—Engorged with blood in lower lobes. Œdematous throughout.

*Heart.*—Left ventricle partially, but not fully contracted, contained fluid blood and small clot. Left auricle contained small amount of clotted blood. Right side of heart contained clotted blood in considerable quantity; clots all dark in color.

*Valves.*—Normal in thickness and competent. Endocardium—Deep wine color; smooth.

*Myocardium.*—Soft, friable. Few spots of emphysema under visceral layer of the pericardium. Left coronary artery contained a clot at distal side of first branch. Intima of artery stained deep wine color.

*Abdomen.*—Peritoneum apparently normal. Gravid uterus with fundus extending to the level of the umbilicus. Large corpus luteum in left ovary. Upon opening the uterus a male fœtus, in the embryonal position, was found. Placenta separated from the uterus by its own weight and without any effort to detach it.

Intestines apparently normal.

Liver enlarged, friable, deeply congested. Spleen slightly enlarged, and very friable. Kidneys congested; acute pyelitis in each pelvis. Bladder normal.

*Brain.*—Some congestion of envelopes, and at posterior margin of *tentorium cerebelli* two small, round, firm tumors intimately attached to the *dura mater* and pressing upon cerebellum at posterior internal angle of each hemisphere. Tumors are each about the size of a filbert, upon section presenting a grayish firm surface at periphery, and a disintegrated portion at the center. Cerebellum.—Soft and pale throughout, *arbor vitæ* appearance almost entirely disappeared. No trace of hæmorrhage or embolism discovered. Ventricles of cerebrum contained little fluid. The *intima* of the vertebral and basilar arteries presented the same appearance as that of the coronary.

(Tumors referred for microscopical examination.)

Professor Jaggard desired to call attention to the condition of the cervix. The cervix is a funnel shaped object, the neck of which measures 4 cm. in length; thickness of wall, 2 cm. The upper, expanded portion measures 1.5 cm. in length; thickness of the wall, 1.5 cm. The mucous membrane lining this funnel-shaped cervical canal differs in its macroscopic characters from the mucous membrane lining the uterine cavity. The cavity of the cervix is filled with a white, coagulated secretion. The insertion of the membranes forms a circle around the upper expanded portion of the cervix, about 7 cm. in diameter, corresponding to the site of several large veins in the muscular substance of the uterus, and the insertion of the peritoneum externally. At this point, the muscular substance of the uterine wall becomes thinner. The average thickness of the muscular wall of the uterus is 1 cm.; that of the cervix 1.5 to 2 cm. Total length of the uterus, 17 cm.

The macroscopical characters of the preparation seemed to sustain the position assumed by Bandl, Küstner and Carl

Braun, recently opposed with considerable force by M. Hofmeier. Dr. John Bartlett, a distinguished Fellow of the Society, read a paper entitled **The Cervix Uteri Before, During and After Labour*, July 14, 1873, before the Chicago Medical Society (several years prior to the appearance of Bandl's classical monograph upon the same subject) from which the following extract is made:

"Early in pregnancy the neck of the uterus is called upon to supply its quota to the enlarging body. Speaking somewhat figuratively, as ring after ring of tissue is demanded from the upper part of the cervix, the preparatory development in the remaining portion is such that the length of the neck is not apparently impaired, so that what remains of it as late as two weeks before labour has been mistaken for the entire infra and supra vaginal cervix, whilst the loss by the continual transfer from the upper portions of the neck to the uterine walls has entirely escaped notice. That circle of the neck which corresponds at the time ot an examination to the limits of its expansion, is regarded by writers as the os internum. The os internum is, of course, as before labor, above the attachment of the vagina, and, near term, far removed from the examining finger. The apparent constriction taken for it is simply that point in the cervical walls marking the constantly decreasing line of demarcation between the expanded and yet unexpanded portions of the neck."

II.—PROFESSOR A. REEVES JACKSON, in beginning the discussion on Dr. Parkes' paper on the treatment of uterine fibroids by ergot, said: I was very much pleased to hear the reading of Dr. Parkes' paper. I commenced using ergot in the treatment of fibroids in June, 1873. I had used it in

---

*The Chicago Medical Journal, October, 1873.

eight cases at the time Dr. Byford read his paper based on 103 observations gathered from various persons in this country. I was extremely pleased with the result; two of the patients seemed to be practically cured—that is to say, while there could be distinguished some remaining enlargement of the uterus, the symptoms that were referable to the presence of the tumor were entirely removed, and the patients suffered no inconvenience from the bulk of the uterus. In nearly every case there was improvement. I continued to use it for several years, but have not used it lately—I do not know why. The cases that have been published by those who use it extensively have all shown favorable results except those of Martin, of Berlin, and perhaps two or three others. There seems to be no reason to doubt that ergot, whether given hypodermically, by the mouth or rectum, does have some controlling influence on the development of uterine myomata, checking the growth or lessening the size of the tumor. Indeed, there is reason to believe that it is one of the very best means of dealing with these tumors. I have used the remedy in perhaps thirty cases. I do not know just what the ratio of success was. In about three-fourths of these cases there was benefit. Sometimes the good effect did not consist in diminution of the size of the neoplasm, but from improvement in the general health of the patient. I was very glad to hear of the almost phenomenal success that followed the practice of Dr. Parkes. In some of the cases he relates the patients were, however, evidently in great jeopardy from the sloughing of the mass, and the difficulty of getting it away before septicæmic symptom came on. There is great danger, unquestionably, in having a sloughing fibroid retained within the uterus. The treatment by ergot should be accompanied by dilation of the cervix, so that the mass, when separated from the wall of the uterus, may escape readily. This would lessen that danger. In some cases death has occurred very soon after the stinking discharge appears. Nevertheless, the treatment by ergot is very much less dangerous than any of our surgical methods of dealing with uterine fibroids.

THE PRESIDENT: Have you kept records of any of your cases?

PROFESSOR JACKSON: Yes, and I shall be glad, if the interest continues, to report them in detail. I kept accurate notes of the first cases so far as I had charge of the patients. Some of them occurred in the Woman's Hospital, and the patients would go away, and we did not always have means of ascertaining the final results. But of others, occurring in private practice, I can give accurate details.

DR. H. T. BYFORD: I made the assertion that there was no danger of a sloughing of the tumor when it is not situated so that it can be expelled by way of the vagina. This was based on the fact that, unless submucous, it cannot be firmly enough compressed. I have reported a case of fibroid tumor of the vagina,* whose thick pedicle was gradually cut through by daily tightening a fine wire about it; when the wire had cut through the pedicle, it was found to have reattached itself and retained its vitality, showing that tumors of this nature require very little nourishment to keep them from undergoing sloughing. The cases on record are very few in which subperitoneal growths have sloughed from the use of ergot.

PROFESSOR W. W. JAGGARD said, with reference to priority in the use of ergot in the treatment of uterine fibroids, that Hildebrandt, of Königsberg, had published a paper in 1872, in which he recommended the drug. The growth of the neo-

* Chicago Medical Journal and Examiner, August, 1885.

plasm was limited by diminished access of blood, and, in some cases, it was actually expelled from the uterine cavity. Hildebrandt's recommendation was the revival of an old practice. Dr. Wm. H. Byford's paper—Address on Obstetrics—was read in Philadelphia in 1875. During the interval of three years, several papers were written extolling the action of ergot in the treatment of uterine fibroids, both in the diminution of the quantity of blood flowing to the tumor, and also in actively causing its expulsion from the uterus.

DR. HENRY T. BYFORD said: Dr. Jaggard fails to take into consideration the different ways in which ergot acts. It acts first, in a radical way by expelling the tumor, as in the submucous variety; second, in a gradual way by causing atrophy and absorption, as in the interstitial variety; third, in a partial way, by arresting the tumor's growth and activity, as the subserous ones; fourth, in a palliative way by relieving the symptoms, as in cases of large tumors, near the menopause. Schroeder in the last edition of his *Krankheiten der Weiblichen Geschlechtsorgane*, gives Dr. Wm. H. Byford credit for suggesting the use of ergot for the expulsion of the tumor. There is no longer any reason to doubt that ergot is the surest and safest cure for all but the very exceptional cases of uterine fibro-myomata. A tolerance of moderate doses is quickly established both by the organs through which it is absorbed and by the general system. Sloughing is almost never produced except in the submucous variety, when it need not be dangerous. For several years past, with one or two exceptions, I have not given ergot in any other way than by the rectum. I use 5 to 8 grains of Squibb's extract of ergot twice a day and continue it for two or three years, with favorable results. I remember one case in which the tumor extended almost to the umbilicus when I first saw her, five years ago. It was an irregular, nodulated tumor, mostly subperitoneal, with projections larger than the fist, filling up the pelvis, and to a great extent the false pelvis also, and sometimes caused excruciating pain by its pressure. The patient had repeatedly bled through six and eight weeks, and must have lost one hundred pounds. Tampons were required to save her life. I never saw paler mucous membranes in a living being. It was a very much worse case than many which I continually find cited in medical literature, in which hysterectomy is considered necessary. The patient begged to have the tumor removed. She could not take the ergot for any length of time either by rectum or mouth; but after a while she tolerated 5-grain rectal suppositories, and has passed the menopause. The tumor, having lost its activity, has become considerably smaller; while she, having regained her hundred pounds, has become considerably larger. I have a case which had been treated for a year with all of the most approved remedies, except ergot. When I first saw the patient, two years ago, she weighed eighty-three pounds; she had a nervous chill and almost fainted when I first entered the room, because I was a stranger. She had not slept for weeks except under the influence of narcotics, and had symptoms of acute tuberculosis. She was in the habit of bleeding steadily from three to six weeks, and was being so rapidly destroyed by the loss of blood that I at first had to use the tampon. She was put upon eight grains of Squibb's fluid extract of ergot per rectum and tincture of iron by the mouth. Her health improved rapidly and the hæmorrhages progressively diminished. Her lungs were recently examined by Dr. H. A. Johnson, who found the remains of old trouble, but no tendency to unfavorable changes. Her cough, which had lasted so long, has entirely left her. She now takes the ergot a part of the time only. Her menses last four days, are natural in quantity and quality, and are followed every two or three months by a watery discharge of a faint pinkish tinge. She cannot feel the tumor now, although a projection the size of a child's head was formerly felt by her between the umbilicus and left groin. It might be said of this case that it was also a very proper one for operation, and one in which ergot, if harmful to the system, would have done injury. I have similarly relieved other cases nearly as bad, and cannot help believing that, when treated early, judiciously and persistently by ergot, fibroid tumors of the uterous will show a mortality of 1 or 2 per centum instead of 10 per centum as at present; that hysterectomy for fibroids, with its mortality of 20 to 40 per centum, will eventually become an interesting relic, and the removal of the appendages a precious rarity.

DR E. W. SAWYER said: There is one point that was not alluded to by the reader of the essay, and that has not been spoken of in the discussion. The fact that the point has been proved in practice shows that it is worthy of attention. That ergot will cause atrophy of a uterine fibroid, causing a detachment by ulceration and expulsion, is a well-established fact. When the fibroid is submucous, or nearer to the mucosa of the uterus than to its peritoneal suface, I have no doubt that that process can be continued and completed with safety. But let us suppose a tumor very close to the peritoneal surface; this process of atrophy takes place, the peritoneum ulcerates through, and the life of the woman is jeopardized. Such a condition occurred in a patient seen by the President of this Society, and it was shown that had the patient lived long enough the fibroid might have been thrown off through the abdominal parietes. This patient died of peritonitis. The large and partially detached fibroid was in a sac containing a great quantity of pus. This sac had ruptured, occasioning the peritonitis.

Professor F. E. Waxham said: I would simply add my testimony as to the value of ergot in the treatment of submucous fibroids, by citing a case: A woman, 45 years old, came to me some time ago complaining of copious hæmorrhages at her menstrual periods, and upon careful examination an enlarged uterus was found, which nearly reached the umbilicus. The diagnosis of fibroid of the uterus was made. This woman was placed upon ergot, combined with opium, to control the pain, which she took for several weeks. Between the second and third months after commencing to take the ergot, I was called to her in great haste and found her apparently in labor, the uterine contractions quite regular and very severe; a partially dilated os and the tumor presenting. This tumor was expelled after two or three hours. It was nearly as large as a child's head at time of birth. The patient made a complete recovery.

The President: In what state was the tumor?

Professor Waxham: I can hardly say it was softened, but it was fleshy in character, and some pus upon it as though it had suppurated. It was somewhat offensive, I remember. I attended her for some weeks subsequently; there was some febrile reaction, but no serious trouble followed.

The President said: I have reported a case in which a tumor was thrown off without sepsis. I have had since quite a series of cases in which tumors have been thrown off; some have been absorbed and there has been a various history, which I hope to make the subject of a special paper, and would like the assistance of others in making up a history of these cases. I think we are specially favored in having with us Dr. Wm. H. Byford, who has had such extended experience in these cases.

PROFESSOR W. H. BYFORD, in closing the discussion, said: Mr. President, you are right in supposing that I feel great interest in this subject. I have made it a study for a long time. Perhaps as good a way as any to introduce my views on this subject to the Society, will be to go back to the commencement of my own researches in this matter. In 1872, as Dr. Jaggard has said, Hildebrandt commenced a series of experiments for checking the hæmorrhages connected with fibroid tumors of the uterus, by giving hypodermic injections of the extract of ergot, and succeeded in a great many instances in suppressing the hæmorrhage and relieving the patient from the inconvenient symptoms. During these experiments he also ascertained that the tumor would sometimes disappear. I think his statistics were not large, and that he only reported a very few, perhaps three or four, cases in which the tumors disappeared by atrophy during the time he treated them in this way. In 1874 I was elected to the chairmanship of the Section of Obstetrics in the American Medical Association, and as these experiments of Hildebrandt had attracted considerable attention, I thought it would be a good time to make some investigations as to the value of his facts. I commenced correspondence over a large portion of the United States and Europe, but especially communicated with my friends in this part of the country, among whom were my immediate associates in this city, who had been engaged in using hypodermic injections of ergotine according to the method of Hildebrandt, once in two or three days. All of them bore testimony as to the efficacy of that kind of treatment, and as to the fact that these tumors could be made to disappear in a great many instances by atrophy, and in a great many more the symptoms could be relieved so that the patient was rendered comfortable, the presence of the tumor giving them but little inconvenience. Some of the gentlemen with whom I had correspondence had been using the ergot in different ways, giving it by mouth, giving it *per rectum*, and injecting it into the tumor itself and by various other methods. I noticed one fact in my own practice and that of my friends, which was that the more frequently the ergot was given the more powerful its action was. In giving it two or three times a week hypodermically by the Hildebrandt method, there is very little distress produced by it; but the tumor may gradually disappear and the symptoms get better. I collected 103 cases from different parts of the country, and in all of them the attention of the practitioner was directed to the point of causing the disappearance of the tumor by atrophy. During the time I was making these investigations cases of fibroid tumors occurred in the practice of my friends, who consulted me. One was a remarkable instance in the practice of Dr. Merriam. I remember the particulars. The patient was a little Irish woman who had a tumor almost large enough to reach to the umbilicus. He commenced the use of ergot in September, 1874, twenty drops of Squibb's fluid extract three times a day. It produced so much contraction of the uterus and so much pain as to alarm the patient and the doctor himself; he thought these pains ought to be suppressed, and as a consequence he would intermit the use of ergot, give anodynes to stop the pain and get relief from the sufferings of the patient, but would recur to ergot as soon as his fears had subsided. In January, 1875, he directed her to recommence the ergot and increase the amount. He gave her, I remember very well, twenty-five drops of Squibb's fluid extract three times a day. In March, which was about two months from the time he began giving her ergot in that way, the patient commenced having expulsive pains very much like labor, and not long after that, probably about March 20th, there commenced to issue from the vagina a putrid liquid that was very offensive and which contained small pieces of organized substance. He became alarmed and entirely withdrew the ergot, supposing he was doing mischief, but the death of the tumor had been produced and as a consequence the uterus continued its action to throw off this foreign body, until April 5, 1875, he was summoned in great haste to see his patient. I was also summoned. Upon arriving at the house, which he did before me, he found the tumor expelled, part of it laid in the vagina and part between the limbs of the patient, a protruding mass almost the size of a child's head. It was not expelled in a lump, but was broken in pieces that would represent that size. The patient at that time had septic fever, with increased temperature and increased frequency of pulse, etc. The Doctor and I both felt uneasy about her, but she very soon rallied and in a short time was well, and since has given birth to a child.

That was my first observation as to expulsion of tumors of that kind. It started a train of thought in my mind and led me to think about increasing the ergot beyond the amount that had usually been given for producing atrophy. In the same year, July, 1875, I commenced giving it with the view of expelling a tumor. I gave my patient at first fifteen-drop doses of Squibb's fluid extract three times a day, and increased it until the patient was taking a teaspoonful of ergot three times a day. On August 15th, about five weeks after I commenced using it, the tumor was broken up and expelled from the vagina. It was expelled by pieces, the first piece about as large as my thumb, of a grayish kind of substance that smelled very badly. The action continued; I was somewhat alarmed and gave the patient anodynes, but the uterus had already commenced to act on the tumor and expelled it, as it would any foreign body. In December of the same year I had an opportunity of repeating the experiment, and the case terminated in the course of six weeks, by the same method of administering the ergot. In 1876, on returning from the world's exposition at Philadelphia, I was requested to call at Coldwater, Mich., to see two patients, one with cancer and one with a tumor. I found one of these patients with a tumor as large as my head, the measurement of the cavity being fully six inches. I told her I believed the tumor could be expelled if she was willing to go through the process. I felt uneasy, however, to leave her to use such medicine by herself, and tried to teach her how she should proceed when the expulsion should take place. She took the ergot three months without much effect, except that occasionally she would have a paroxysm of pain; after that, however, the pains became so very severe that she could not take the ergot much of time. But, brave and intelligent as she was, she repeatedly resumed it, and finally the tumor commenced to come away. It came away in about five weeks from the time the first symptoms of expulsion occurred. She wrote me a description of the method of expulsion. She said at first small lumps made their appearance and passed out of the vagina; after the second day they became larger, and on the third and fourth days they seemed large enough to fill up the vagina. With her scissors she cut off pieces of it and pulled at it to assist its removal. She labored at it two or three days until it was all expelled. In about three weeks thereafter she came to see me, and the uterus had shrunk back to near its natural size. She has since had the menopause, and is now in good health. She sent me at that time a quart cup full of this expelled fibrous substance.

Another case occurred in the western part of this state, under the care of Dr. Crandall, of Sterling. The patient came, by his directions, to see me, and I found a tumor of considerable dimensions and advised her to take ergot. She went home and in about fifteen or twenty days got her work done up, as she expressed it, took three doses of thirty drops of Squibb's fluid extract of ergot, and started up such a process of expulsion that, notwithstanding the efforts of her physician to stop it, the pains went on to the expulsion of the tumor, which was completed in about three weeks.

Dr. Wm. Fox, of Milwaukee, three years ago sent me a report of another similar case. In summing up these observations, I have known personally of twenty-six cases of expulsion of the tumor in this way. With reference to the dangers connected with the expulsion, I would say that only one out of these twenty-six cases proved fatal. They all had septicæmia to some extent, but as soon as the mass of dead tumor was removed the patient commenced to recover and got well. Some of the patients had no assistance. This one patient in whom it proved fatal lived in Monmouth. It occurred about six years ago. She was a lady who, like all other foolish women, distrusted her home physicians, and she came here supposing she would find better treatment. I advised her to take ergot, and in about three months the pains commenced that caused the tumor to be expelled. She came here with the lower part of the tumor hanging from the vagina and uterus while the upper portion was clinging to the cavity in which the whole of it had been lodged. She was then laboring under a high fever. The smell was terrible. She came to the Tremont House and it was several hours before I could see her. When I arrived it was a very simple matter to enucleate it, and I removed it in a few minutes. But she had already received a fatal poisoning from the retention of the dead tumor. This is the only case I have known to prove fatal. I do not get a history from other gentlemen of any more unfavorable results. They all tell me they are frightened at the symptoms, and they are afraid the patients are going to die, but they do not die. When the mass is taken away and the vagina washed out the symptoms disappear. Since thinking of this matter and observing the effects of this remedy I have thought that I could come to definite conclusions as to the conditions under which we might predict the expulsive effects of ergot by the appearance of the tumor. You know that it is not a very common thing to find a case in which there is a single tumor in the fibrous tissue of the uterus. More frequently these tumors are complex. Quite a number of nuclei of formation; we often see in one uterus four or five, sometimes fifty different points of solidification. Now a single, or even a double tumor, located within the circle of the fibrous arch of the uterus near the mucous membrane, is the kind that I think may almost certainly be expelled. If you find a case of symmetrical development, where the uterus seems near its normal shape, no matter how big, so it is normal in shape, oval, or globular, without any large projections standing out in various directions, feeling somewhat elastic to the touch, and attended with hæmorrhage, you may be pretty sure you can expel the tumor by commencing with small doses of ergot and increasing them in size, and then when the pains begin, not to stop them. The presence of severe pains frightens a great many men from finishing what they have begun. If I were to try to explain this operation I would say when ergot is given in this way, after a while the tumor becomes starved, the supply is cut off so there is not blood enough to support it, and very soon it dies in consequence of this strangling process. When it dies there is, at the same time, gangrene of the mucous membrane covering it; then it becomes a foreign body and you cannot keep the uterus from expelling it. The expulsion is a consequence of this starvation and killing process in the tumor. As to the action of ergot in tumors that are not sub-mucous, of course I know that tumors not sub-mucous cannot be expelled. There is what is called the interstitial tumor, developed in the central stratum of the fibrous walls of the uterus; these are the proper subjects of the Hildebrandt process for atrophization. Then with reference to the effect of ergot upon subperitoneal tumors; I have often been asked the question, Can ergot affect these subperitoneal tumors? I think they are frequently starved out and cured; when not too near the peritoneum there is no danger of their becoming detached and putrid in the peritoneal cavity, because the action is from the tumor. In the submucous tumor the contractions are all towards it and none from it. There is one circumstance to be taken in connection with these tumors and the action of ergot upon them, that has not been sufficiently considered. A large proportion of them growing to any considerable size contract attachments to the peritoneal membrane, the intestines, omentum, or the walls of the abdomen, and in making this attachment they get a new supply of blood, which makes the life of the tumor more tenacious than it would be otherwise. This very process of adhesion to the walls of the abdomen is, more than any other, the cause of their great size and the change from a fibrous to a fibro-cystic tumor. We need not expect such tumors to be affected by ergot. There are a good many other things that interfere with the successful use of ergot, of which I cannot now speak. I am grateful to my western associates who have assisted me by facts and experiments on this subject. If you go to the eastern part of the United States they will tell you that ergot is of no use in the treatment of fibrous tumors, or it is too dangerous; the patient cannot live under the pains of expulsion, etc.; but if these same gentlemen had a patient in labor they would urge the pains instead of stopping them. Most physicians who do not believe in the efficacy of ergot use Hildebrandt's method pretty much altogether, which produces tonic contraction of the fibres of the uterus, but does not go to the extent of causing expulsive pains. Then, again, there is too great apprehension on the part of the profession generally of the dangerous toxæmia from ergot. I do not know whether the history we have of the poisonous influence of ergot in producing nervous diseases, gangrene, and so on, is true; whether the observations that led to that teaching were correct at one time or not, but I know that after the use of ergot persistently for two or three years in the same case, I have never seen any evil influence produced by it, unless it is in cases where the violent action of the uterus would be regarded as such. I have purposely avoided saying anything about the *modus operandi* of ergot in causing contractions in the uterine fibres, because that is now sufficiently understood by the profession. But, Mr. President, I feel that I have occupied too much of the valuable time of the society already, and will say no more.

DR. H. T. BYFORD read a paper entitled

III.—A STUDY OF THE CAUSE AND TREATMENT OF PELVIC HÆMATOCELES.

The author cited the case of a non-suppurating, retro-uterine hæmatocele of six months' standing, which he evacuated *per vaginam* March 18, 1886, and then treated with antiseptic irrigations. She was up and about the house in eleven days. As the odor and discharge were still causing discomfort, the doctor, influenced by the advice of Apostoli and Doléris, curetted the cavity. He found no more blood or débris, but started up a mild attack of local peritonitis, which delayed instead of hastening the cure. The patient left the hospital in a little less than a month after the cessation of all discharge. A small lump of induration extending from the abscess opening to the right sacro-uterine ligament was all that was left of the tumor.

The following résumé of interesting points in the case is given:

1. The length of time from the occurrence of the hæmatocele to the time of operation, about six months.

2. The method of opening the cavity, viz.: by first tearing the vaginal wall, and afterwards the sac wall.

3. The absence of fluid in the tumor.

4. The breaking up of the mass with the finger without an attempt at thorough curetting or removal of the entire contents.

5. The complete disintegration and discharge of all bloody substance in thirteen days.

6. The absence of high temperature—102° F. having never been reached.

7. The small amount of anodyne required—one dose (except the two doses to relieve the irritation from subsequent unnecessary curetting).

8. The toleration of strong antiseptic solutions. It was necessary to weaken them on account of their effect upon the vagina.

9. The absence of the usual amount of odor in such decomposing masses.

10. The large quantity of food taken throughout.

11. The absence of any kind of sickness from the beginning until the cavity was curetted.

12. The curetting of the cavity on the thirteenth day delayed her recovery, producing the only serious symptoms that were noticed.

13. Notwithstanding a setback of ten days, caused by the curetting, she was well enough to go home inside of a month and dispense with treatment.

14. The attack came on after a miscarriage.

P. F. Mundé reports two new cases of hæmatoma successfully operated upon three and six weeks, respectively, after their occurrence, both large, and resulting from or after abortions. (*N. Y. Medicinische Presse*, Vol. 1, No. 1, Dec., 1885.)

Five other cases were briefly related, four extra-peritoneal hæmatomas and one large retro-uterine hæmatocele, which had come under the writer's observation during the past two years, and which were successfully treated on the expectant plan. He was unable to find justification in any text-book for having operated in the absence of any threatening symptoms until he procured the last edition of Billroth & Lücke's "*Frauenkrankheiten*" and Schroeder's text-book (both of 1886). He cautions against taking the advice of Bandl, to operate after the first subsequent menstrual period, or that of Apostoli and Doléris, to operate immediately by the galvano-puncture wherever and whenever found. Operations at such times are connected with what are designated as immediate dangers, viz.

"a recurrence of shock, hæmorrhage, or (if hæmostatic tampons be used) of inflammation; or of septicæmia followed by inflammation, if antiseptic injections of sufficient strength be used."

The dangers of the expectant treatment are mostly remote, and are such as "suppuration, septicæmia, perforation, and prolonged pressure upon, and displacement of, surrounding organs, with their results, viz.: the aggravation and perpetuation of pre-existing pelvic disease, or the originating of new ones."

While recognizing the necessity for evacuation within the first three or four weeks in certain exceptional cases, he would, as a rule, delay operating long enough to avoid the immediate dangers, yet not long enough to incur the remote dangers of delay. The patient must invariably be kept in bed and the tumor should be left alone until the primary acute symptoms subside. If the tumor remain hard and diminish in size, no matter how slowly, it should be let alone as long as the symptoms do not become worse. If the tumor remain stationary and boggy to the feel, and the symptoms begin, after a few weeks, to increase in severity, it must be operated upon ; or if the symptoms remain without improvement, while the tumor shows no signs of being absorbed, it is better not to wait for serious symptoms, but operate in the subacute stage, or when the symptoms have subsided as much as they will. There is a certain class of cases, like the first one reported, in which the acute symptoms subside, the patient recovers considerable strength, but the tumor remains elastic, or boggy, and almost stationary, and interferes with her usefulness. If the patient cannot, from adverse circumstances, or does not wish to make an invalid of herself for the many months of quiet and carefulness requisite for safe absorption of the organized clot, she should have the benefit of an operation at a time when it is almost devoid of danger.

DR. BYFORD would not select the method of operating recommended by Apostoli and Doléris because two sittings would be required for a complete operation, and because the use of the currette through so small an opening as would be justifiable by galvano-puncture is not devoid of danger. He prefers puncturing and tearing with a dilator, first the vaginal and then the cyst wall, to Zweifel's method of incising, on account of the less liability to trouble from hæmorrhage. He also advises the attack of such retrouterine hæmatoceles as are accompanied by obliteration of the Douglas cul-de-sac by puncture and dilation per rectum, when possible. The difficulty would be but little greater than the dilatation of the fistulous opening of a pelvic abscess.

Though curetting is condemned as dangerous, but a breaking up of all solid material by the finger, and the trusting to copious, strong, antiseptic irrigations (which can be endured much stronger if the abscess walls are not scraped) is recommended. Hydrarg. bin-iodide $\frac{1}{3000}$, or bi-chloride $\frac{1}{2000}$, or carbolic acid 1½ to 2 per cent. twice or three times a day, as necessary. Instead of a drainage-tube being used, the finger may be passed through the opening daily in order to dilate and insure free discharge. On account of diagnostic difficulties an aspirating needle should precede the use of the knife.

The discussion of Dr. Byford's paper was deferred until the next regular meeting.

W. W. JAGGARD, M. D., Editor.

2330 Indiana Ave., August 15, 1885.

